# Harnessing sponge gourd: an alternative source of oil and protein for nutritional security

**DOI:** 10.3389/fnut.2023.1158424

**Published:** 2023-05-16

**Authors:** Ruchi Tyagi, Rakesh Bhardwaj, Poonam Suneja, Amish K. Sureja, Anilabh D. Munshi, Lalit Arya, Amritbir Riar, Manjusha Verma

**Affiliations:** ^1^Bioscience and Biotechnology Department, Banasthali University, Banasthali, Rajasthan, India; ^2^Germplasm Evaluation Division, ICAR-NBPGR, New Delhi, India; ^3^Division of Vegetable Science, ICAR-IARI, New Delhi, India; ^4^Division of Genomic Resources, National Research Centre on DNA Fingerprinting, ICAR-NBPGR, New Delhi, India; ^5^Department of International Cooperation, Research Institute of Organic Agriculture FiBL, Frick, Switzerland

**Keywords:** protein, fatty acids, sponge gourd (*Luffa cylindrica* R.), seed, oil and nutrition

## Abstract

*Luffa cylindrica* (L.) Roem. is an important cucurbit crop that assures food security and dietary diversity among the poor communities. In the present study, seeds of 42 genotypes of *Luffa cylindrica* were evaluated for their potential use as oil seed crop. Seed moisture, oil and protein content and fatty acids profile were estimated along with total phenol and sugar content. Significant differences were observed among the various genotypes where oil content ranged from 15.4–29.8% and protein 19.9–30.8%. Total phenol content was high 6.43–12.84 mg/100 g, which bodes well for the sponge gourd seeds’ ability to act as antioxidants. Significant correlation were found between important constituents studied like protein and oil, palmitic acid, stearic acid and oleic acid. Total unsaturated fatty acids were in higher amount comparable to saturated fatty acids signifying the good quality of *Luffa* seed oil. Our research revealed that the NDSG-1, Pusa Sneha, DSG-95, DSG-98, DSG-108, and DSG-26 genotypes were very nutritious due to their high levels of protein, oleic acid, and oil output. Additionally, selection of traits having considerable correlation will be beneficial and help in improved varietal development for usage as an alternative oilseed crop. Our research sheds light on the nutritional value of sponge gourd seeds and suggests using them as a potential source for oil and protein, particularly in underdeveloped countries.

## 1. Introduction

India is one of the world’s 12 centers of crop plant diversity, and its gene pool includes 320 species of wild relatives in addition to 166 species of agro-horticultural crop plants that are dispersed across eight agroecological zones (1, 2). The long history of agriculture and the ethnic diversity on the subcontinent have been significant factors in the diversification of crop resources, leading to the accumulation of rich genetic diversity in a number of crop species and their wild ancestors in this area. The Sanskrit term “Koshataki” denotes the plant’s early cultivation history in India.

Vegetable crops known as cucurbits are members of the Cucurbitaceae family, which is mostly made up of species that are eaten as food all over the world. About 118 genera and 825 species make up the family. Despite the fact that the majority of them were Old World originators, numerous species originated in the New World and at least seven genera were found in both hemispheres. Within the family, there is a great deal of genetic variation, and cucurbit species can thrive in temperate, arid deserts, tropical, and subtropical climates. The genetic variety of cucurbits includes both vegetative and reproductive traits, as well as a wide range in the number of monoploid (x) chromosomes, including 7 (*Cucumis sativus*), 11 (*Citrullus* spp., *Momordica* spp., *Lagenaria* spp., *Sechium* spp., and *Trichosanthes* spp.), 12 (*Benincasa hispida*, *Coccinia cordifolia*, *Cucumis* spp. other than *C. sativus*, and *Praecitrullus fistulosus*), 13 (*Luffa* spp.), and 20 (*Cucurbita* spp.) (3). India’s various agroecological and phyto-geographical regions are incredibly diverse, and many domesticated and wild cucurbit species are thought to have originated on the subcontinent (4) and the primary center of origin of crops such as smooth or sponge gourd (*L. cylindrica* M. Roem.), ridged gourd [*L. acutangula* (L.) Roxb.] and pointed gourd (*Trichosanthes dioica* Roxb.) (5) particularly in the eastern peninsula tracts, Indo-gangetic plains and north-eastern area (6). In India’s north-eastern region, *Luffa* sp. are growing in their natural habitat. Peninsular India is home to *L. acutangula* var. *amara*, while the western Himalaya and upper Gangetic plains are home to *L. echinata*. *L. graveolens*, another crucial species, is found in Tamil Nadu, Sikkim, and Bihar (7).

Through introgression and selection from wild forms that exist in various regions of the country, many species of *Luffa* must have gradually evolved. These species or landraces have useful genes that are adaptable to a wide range of agroecological zones and have resilience to stress, diseases, and pests (6). Recently, sponge gourd genome was sequenced and it was found its genome size sponge was 656.19 Mb which is substantially larger than that of most other sequenced cucurbitaceous species (269–469 Mb) (8). Further phylogenetic analysis allowed the divergence times between sponge gourd genes and their homologs in the other plants to be estimated, indicating that the sponge gourd lineage diverged from the bitter gourd lineage (*M. charantia*) approximately 41.6 million years ago, with subsequent divergence from other cucurbitaceous plants occurring approximately 32.5 million years ago (8).

Sponge gourd [*Luffa cylindrica* (L.) Roem.], is a herbaceous vine of Cucurbitaceae family. The cross-pollinated crop *Luffa* is a diploid species with 26 chromosomes (2*n* = 26) (9). When completely grown, *Luffa* produces tasty green fruits with a cylinder form that can be used as sponges. The young immature fruits and leaves can be prepared as curry or eaten fresh or dry. According to one study, there were significant differences between wild and domesticated species of *Luffa* in terms of their morphological (seed size, color, surface of the seed coat, and 100-seed weight) and biochemical (oil and protein) characteristics (10). They recommended investigating the potential of this valuable crop as a source of edible oil, food, and fodder or as a source of industrial oil/biodiesel. The proximate composition and mineral contents as well as the levels of tannin, oxalate, phytin phosphorus, and phytic acid of *L. cylindrica* were examined. The findings suggested that it may have application as a source of vegetable protein in the diets of both animals and people (11). The oil content and quality features of *Luffa* seeds were evaluated, and this research demonstrated that *Luffa* seed oil is a semi-drying oil that may be employed in surface coating applications such paints, resins, and printing inks. Additionally, seed oil has the potential to be used as a feedstock for the manufacture of soap and biodiesel (12).

In the past, studies have been carried out in India to highlight the genetic diversity of *Luffa* based on morphological data and molecular markers (13–19). *Luffa cylindrica* has chemicals that affect hypersensitive reactions, act as immunostimulants, anti-inflammatories, and participate in glycosidase activity. They also decrease protein synthesis with type I RIPs’ structural-function relationships suggesting potential for antitumor and antiviral activities. Finally, they promote uterine contraction to speed up labor (20). Due to abundance of unsaturated fatty acids (80%), as well as the primary fatty acids like linoleic acid (60–65%) and oleic acid (15–20%), in seed oil, tests on mice showed an increase in high density lipoproteins (21).

Additionally, the sponge gourd’s leaves, seeds, and fruits display remarkable therapeutic properties, including anti-inflammatory, analgesic, anticancer, hepatoprotective, antibacterial, and wound healing action, and the triterpenoids (sapogenins 1 and 2) that were derived from the sponge gourd exhibit immunomodulatory effects (22–27).

Seeds can be used to cure illnesses like leprosy, sinusitis, and others because they contain a variety of phytochemicals that have the ability to heal wounds and kill bacteria (28, 29). The seeds have alcalase or tryptic protein hydrolysates that are useful for treating diabetes and hypertension (30). Because sponge gourd has 462 NBS-LRR genes, which are involved in nucleic acid metabolic and defense response processes, it has higher stress resistance than other cucurbitaceous species and is frequently used as a rootstock in bitter melon and bitter gourd to increase crop yields, combat soil-borne diseases, and improve flooding tolerance (31, 32).

Due to its antifungal, anti-inflammatory, and anti-tumor qualities, seed oil is utilized in the cosmetics industry (33). The presence of luffin, a ribosome-inactivating protein that prevents the growth of HIV and other diseases, may be the cause of the therapeutic benefits of seeds (34). Despite being poisonous and bitter, seed cake can be utilized as fertilizer due to its high levels of phosphate and nitrogen (35).

Sponge gourd is considered as an emerging income crop due to its several uses as a food, medicine, bath sponge, seed oil, and seed protein (36). The mature fruits of sponge gourds are increasingly prized for their exceptional seeds and high-quality sponge. Standardized solvent extraction techniques have been developed for oil extraction from sponge gourd seeds (37). These findings give a justification for the amazing medical potency of sponge gourd, which has garnered contemporary scientific interest. Nevertheless, there is not much information about this crop’s nutritional makeup.

For this reason, it is crucial to examine the nutritional makeup of *Luffa cylindrica* samples gathered from various geographic locations. With the aforementioned information in mind, the objective of the current study was to assess the genetic diversity of 42 genotypes of sponge gourds collected from various Indian regions for quality traits of seeds in order to select the best parent for crop improvement. The quality traits included phenol content, protein, oil, and fatty acid profile.

## 2. Materials and methods

### 2.1. Plant material

**Figure 1:**
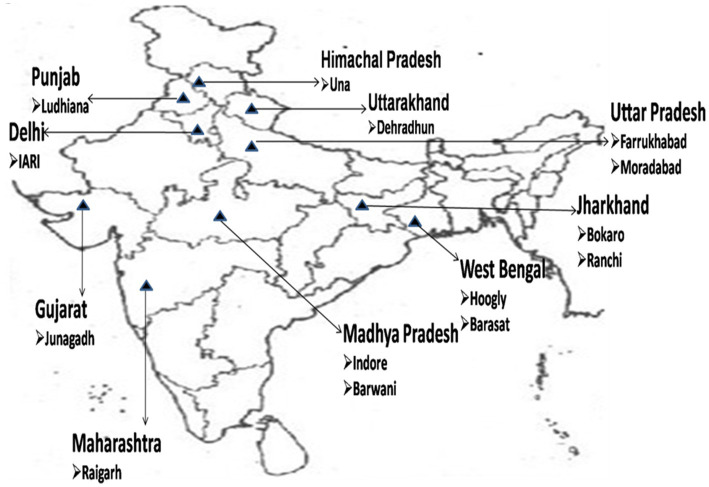
Representation of different geographical regions of India from where samples were collected.

The study used 42 sponge gourd genotypes collected from various parts of India and maintained as inbreeds at the Division of Vegetable Science, ICAR-Indian Agricultural Research Institute (IARI), New Delhi (Table 1; Figure 1). The experiment was set up in a randomized block design with three replications during the spring and summer of 2021–2022, with an average temperature of 26°C, an average relative humidity of 46.1%, and average effective precipitation of 5.7 mm and a 14 h light/10 h dark cycle, respectively. The soil has 109.0 Kg ha^–1^ of nitrogen and 9.05 Kg ha^–1^ of phosphorus, respectively. The lines were sown in rows of 2.5 m with 75 cm spacing between the plants, with 15 plants per replication. All the plants were grown at the same time and in same location. All cultural practices recommended for the successful cultivation of the crop were followed. Fruits are harvested when the skin has changed from green to brown or yellowish-brown, which typically takes 150 days. Observations were made on 10 plants at random in each replication. Fruits from each row were composited to make single replicate. Harvested fruits were oven dried at 60°C for 72 h and seeds were collected from dried fruits.

**Table 1:** Description of place of origin of sponge gourd genotypes along with their geographical locations and mean annual temperature (MAT).

S. No.	Genotype name	Place of origin	Latitude	Longitude	MAT
1	DSG-6	Hoogly, West Bengal	22°53′60″	88° 23′24″	30.44
2	Pusa Supriya	IARI, New Delhi	28°38′23″	77°10′10″	25.0
3	DSG-7	Moradabad, Uttar Pradesh	28°49′53″	78°46′42″	24.1
4	VRSL-1	Hoogly, West Bengal	22°53′60″	88° 23′24″	30.44
5	VRSL-2	Hoogly, West Bengal	22°53′60″	88° 23′24″	30.44
6	VRSL-4	Hoogly, West Bengal	22°53′60″	88° 23′24″	30.44
7	VRSL-5	Hoogly, West Bengal	22°53′60″	88° 23′24″	30.44
8	VRSL-6	Farrukhabad, Uttar Pradesh	27°22′58″	79°35′30″	30.22
9	VRSL-7	Farrukhabad, Uttar Pradesh	27°22′58″	79°35′30″	30.22
10	VRSL-8	Farrukhabad, Uttar Pradesh	27°22′58″	79°35′30″	30.22
11	VRSL-9	Farrukhabad, Uttar Pradesh	27°22′58″	79°35′30″	30.22
12	VRSL-10	Farrukhabad, Uttar Pradesh	27°22′58″	79°35′30″	30.22
13	VRSL-11	Moradabad, Uttar Pradesh	28°49′53″	78°46′42″	24.1
14	VRSL-12	Moradabad, Uttar Pradesh	28°49′53″	78°46′42″	24.1
15	VRSL-13	Moradabad, Uttar Pradesh	28°49′53″	78°46′42″	24.1
16	VRSL-14	Moradabad, Uttar Pradesh	28°49′53″	78°46′42″	24.1
17	VRSL15	Moradabad, Uttar Pradesh	28°49′53″	78°46′42″	24.1
18	NDSG-1	Faizabad, Uttar Pradesh	26°46′12″	82° 9′0″	24.0
29	PSG-9	Ludhiana, Punjab	30°54′11″	75°51′21″	23.5
19	DSG-31	Barwani, Madhya Pradesh	22°1′48″	74°54′0″	29.93
20	CHSG-1	Ranchi, Jharkhand	23°20′38″	85°18′34″	27.24
21	CHSG-2	Ranchi, Jharkhand	23°20′38″	85°18′34″	27.24
22	DSG-43	Hoogly, West Bengal	22°53′60″	88° 23′24″	30.44
23	DSG-48	Barasat, West Bengal	22°43′22″	88°28′50″	30.4
24	Pusa Sneha	IARI, New Delhi	28°38′23″	77°10′10″	25.0
25	HASG-5	Ranchi, Jharkhand	23°20′38″	85°18′34″	27.24
26	PSG-93	Ludhiana, Punjab	30°54′11″	75°51′21″	23.5
27	PSG-100	Ludhiana, Punjab	30°54′11″	75°51′21″	23.5
28	PSG-110	Ludhiana, Punjab	30°54′11″	75°51′21″	23.5
30	NSG-1-11	Raigarh, Maharashtra	18°30′56″	73°10′55″	24.75
31	NSG-28	Raigarh, Maharashtra	18°30′56″	73°10′55″	24.75
32	JSLG-55	Junagadh, Gujarat	21°31′19″	70 27′28″	25.7
33	DSG-47	Firozabad, Uttar Pradesh	27°9′32″	78 23′44″	24.0
34	Improved Chikni	IARI, New Delhi	28°38′23″	77°10′10″	25.0
35	DSG-95	Una, Himachal Pradesh	31°28′6″	76°16′14″	21.6
36	DSG-98	Dehradun, Uttarakhand	30°18′59″	78°1′55″	20.4
37	DSG-104	Dehradun, Uttarakhand	30°18′59″	78°1′55″	20.4
38	DSG-108	IARI, New Delhi	28°38′23″	77°10′10″	25.0
39	DSG-26	Hoogly, West Bengal	22°53′60″	88° 23′24″	30.4
40	DSG-30	Bokaro, Jharkhand	23°40′9″	86°9′4″	29.77
41	DSG-32	Barwani, Madhya Pradesh	22°1′48″	74°54′0″	29.93
42	DSG-34	Indore, Madhya Pradesh	22°43′10″	75°51′27″	25.3

MAT (mean annual temperature) was computed using information from weather stations located in the areas where samples were taken.

### 2.2. Seed and oil yield

Fully matured dried fruits were harvested at regular intervals, the seeds were extracted and seed weight was recorded. Seed yield per unit area was recorded in Kg ha-1. Seed yield of each replicate was multiplied by oil percentage to calculate oil yield.

### 2.3. Sample preparation for biochemical analysis

Fifty gram seeds in each of the 42 genotypes were homogenized by using a stainless-steel mixer grinder. Fine powder was prepared by grinding and then sieved using a test sieve of ASTM 35 to ensure homogeneity and kept at − 20°C till further investigation.

### 2.4. Moisture content

Five gram sample was dried in oven at 110°C overnight and constant dry weight (DW) was attained. The equation used for moisture content determination was $100\times \left[\left(FW-DW\right)/FW\right]$ and presented in percentage.

### 2.5. Ash content estimation

Five gram of dried ground sample was taken in silica crucible and initial charring was done at 250°C for 1 h and then the temperature was raised to 450°C and retained for 2 h. For complete ashing ash was moistened with double distilled water and 2–3 drops of concentrated HNO_3_ were added. Crucible were again kept in muffle furnace at 450^°^ C for 30 min. Ash content was presented in g/100 g fresh weight.

### 2.6. Crude fat estimation

Extraction of 10 g of homogenized sample was done with petroleum ether at 40–60°C (38). Extraction was performed for 24 h and overnight drying was done at 60°C till constant weight before and after extraction. Food reference material AS-FRM 6 (fish meal 2) provided by Institute of Nutrition, Mahidol University, Thailand was used and recovery of 95.8 ± 3.6% was obtained.

### 2.7. Estimation of total protein

Total protein was estimated with some modifications (39). Hundred mg of dried and homogenized sample was digested and estimated for nitrogen content as per (40) using sulfuric acid–selenium–anhydrous sodium sulfate–hydrogen peroxide digestion mixture. To ascertain recovery, food reference material AS-FRM 14 (provided by Institute of Nutrition, Mahidol University, Thailand) was used as a control. The recovery percentage of 98.9 ± 1.9 for AS-FRM 14 was obtained.

### 2.8. Soluble sugar and total phenol extraction

Hundred mg of homogenized flour was extracted with 5.0 ml of 80% ethanol in an ultrasonic bath at 70°C for 60 min. The contents were centrifuged at 5,000 g for 20 min. The residue was extracted thrice with 5.0 ml of 80% ethanol and supernatants were pooled and volume was made up to 25 ml. This supernatant was kept at − 20°C in the dark.

### 2.9. Total soluble sugar estimation

The total soluble sugar in the sample’s 80% ethanolic extract was calculated by anthrone reagent method (41) with minor modifications as mentioned in Padhi et al. (42). Briefly 100 μl of the extract was evaporated in test tubes on a water bath until dry. The residue was dissolved in 1.0 ml of water and 4.0 ml of anthrone reagent was added. The absorbance was read at 660 nm and calibrated against sample blank and dextrose as standard.

### 2.10. Phenols estimation

The Folin–Ciocalteu Reagent (FCR) method was used to evaluate the phenol content of the ethanolic extract using gallic acid (0–100 g/ml) by method of (43) with minor modifications as described (42). Before extraction, samples were spiked with known concentrations of the standard to determine recovery. The recovery rate was 98.7 ± 1.2.

### 2.11. Estimation of fatty acids

*Luffa* seed samples were freshly ground (with a Remi homogenizer) and weighed so that 40 mg of oil could be extracted using a solvent mixture of chloroform, hexane, and methanol (8:5:2 *v*/*v*/*v*) in 10 ml. The resulting extracts were dried for 30 min. at 60°C in nitrogen gas and methyl esters of oil samples were prepared with a few minor modifications (44) as applied to Cucurbitaceous species (45). 1 μl of the derivatized hexane extract was injected onto a highly polar HP Innowax capillary column that was 30 m long (inner diameter: 0.32 m, film thickness: 0.5 μm, split: 1:80). The gas chromatograph in question was a Hewlett Packard model 6,890 one with a flame ionization detector (FID). Temperatures for the injector and detector were 260°C and 275°C, respectively. The temperature of the oven was designed to rise from 150°C holding for 1 min to 210°C at a rate of 15°C/min, then from 210°C to 250°C at a rate of 5°C/min for 12 min.

Fatty acid methyl esters peaks were identified by comparing the retention times of fatty acid peaks with those of FAMES standard mixture done on 30 m HP Innowax column while running under similar separation circumstances using HP3398A software.

### 2.12. Data analyses

The data were subjected to statistical analysis program SPSS v. 16 (In., Chicago, IL, United States) to calculate mean, standard deviation (S.D.), one-way ANOVA (analysis of variance) as well as the significance of the difference between the mean by Duncan’s multiple range test (*p* < 0.05). After normalizing the data with eigenvalues > 1, principal component analysis (PCA) was carried out to identify the primary components causing the variation in the data. Additionally, the Pearson’s correlation between several quality parameters was established. The link between different accessions based on various features was also determined by creating a dendrogram using Ward’s approach based on Euclidean distance. Values are presented as the average over three replicates ± the standard deviation (S.D.).

## 3. Results

### 3.1. Seed and oil yield

Table 2 shows the seed and oil yield data of *L. cylindrica*. The seed yield ranged from 334 to 600 Kg ha-1 while oil percentage varied from 15.39 to 29.77%. Oil yield was calculated as the product of oil percentage and seed yield, which ranged from 58.1 to 136.9 Kg ha^–1^ with an average of 91 Kg ha^–1^. However, there was a negative association between seed yield and oil percentage. Hence, the accession with the highest seed yield, i.e., DSG43 was not the highest in oil yield. The highest oil yield potential was observed in DSG108.

**Table 2:** Seed and oil yield of 42 genotypes of sponge gourd.

Genotypes	Seed yield (Kg ha-1)	Oil yield (Kg ha-1)
DSG-6	415 ± 12.6	69.3 ± 2.1
Pusa Supriya	531 ± 16.2	98.1 ± 3.0
DSG-7	392 ± 11.9	80 ± 2.4
VRSL-1	363 ± 11.1	58.1 ± 1.8
VRSL-2	375 ± 8.2	79 ± 1.7
VRSL-4	346 ± 7.5	72.3 ± 1.6
VRSL-5	450 ± 9.8	97.6 ± 2.1
VRSL-6	392 ± 8.6	80.0 ± 1.7
VRSL-7	369 ± 14.4	75.3 ± 2.9
VRSL-8	334 ± 13.1	74.6 ± 2.9
VRSL-9	375 ± 14.7	91.5 ± 3.6
VRSL-10	392 ± 15.3	84.3 ± 3.3
VRSL-11	369 ± 14.4	79.7 ± 3.1
VRSL-12	346 ± 10.5	103.1 ± 3.1
VRSL-13	404 ± 12.3	86.0 ± 2.6
VRSL-14	381 ± 11.6	86.4 ± 2.6
VRSL-15	369 ± 11.2	81.9 ± 2.5
NDSG-1	548 ± 21.4	120.0 ± 4.7
PSG-9	484 ± 18.9	90.1 ± 3.5
DSG-31	577 ± 22.5	88.8 ± 3.5
CHSG-1	450 ± 17.6	96.7 ± 3.8
CHSG-2	484 ± 18.9	95.9 ± 3.7
DSG-43	600 ± 13.1	97.8 ± 2.1
DSG-48	565 ± 12.3	104.3 ± 2.3
Pusa Sneha	554 ± 12.1	122.9 ± 2.7
HASG-5	450 ± 9.8	86.8 ± 1.9
PSG-93	405 ± 7.3	76.5 ± 1.4
PSG-100	428 ± 7.7	101.9 ± 1.8
PSG-110	440 ± 7.9	90.5 ± 1.6
NSG-1-11	497 ± 9.0	101.5 ± 1.8
NSG-28	463 ± 8.3	78.2 ± 1.4
JSLG-55	393 ± 10.4	87.3 ± 2.3
DSG-47	428 ± 11.3	90 ± 2.4
Improved Chikni	486 ± 12.8	76.3 ± 2.0
DSG-95	532 ± 14.1	105.4 ± 2.8
DSG-98	484 ± 14.7	106.6 ± 3.2
DSG-104	507 ± 15.5	111.1 ± 3.4
DSG-108	496 ± 15.1	136.9 ± 4.2
DSG-26	438 ± 13.3	127.7 ± 3.7
DSG-30	416 ± 11.0	98.7 ± 2.6
DSG-32	393 ± 10.4	79.0 ± 2.1
DSG-34	521 ± 13.7	126 ± 3.3

Values are means ± s.d.

### 3.2. Biochemical analysis

The biochemical parameters with the lowest variability included moisture content (4.35–7.51%) and ash content (1.25–5.17%; Table 3). Two traits, sugar and oil, showed the biggest variability. The sugar concentration varied between 11.91 (PSG-9, Punjab) and 26.43% (CSHG-2, Jharkhand), with 14.52% being the largest difference. Significant variation was seen in the sugar content of the genotypes from Uttar Pradesh, which ranged from 14.57 (VRSL-8, Uttar Pradesh) to 23.63 (VRSL-12, Uttar Pradesh). While the sponge gourd’s seed oil content varied from 15.39 (DSG-31, Madhya Pradesh) to 29.77% (VRSL-12, Uttar Pradesh). Moderate variations were found in protein (20.17–27.67%) and phenol content (6.49–12.85 mg 100 g^–1^) respectively.

**Table 3:** Nutritional composition of 42 genotypes of sponge gourd.

Genotypes	Moisture (%)	Ash (%)	Protein (%)	Sugars (%)	Phenol (mg100g-1)	Oil (%)	SFAs (%)	PUFAs (%)
DSG-6	5.21 ± 0.07	4.41 ± 0.04	23.88 ± 0.79	21.42 ± 1.73	7.45 ± 0.10	16.67 ± 1.29	19.12 ± 0.21	80.885 ± 0.21
Pusa Supriya	4.91 ± 0.12	5.17 ± 0.15	22.83 ± 1.02	18.71 ± 0.64	7.29 ± 0.09	18.45 ± 0.73	17.24 ± 0.35	82.76 ± 0.34
DSG-7	7.51 ± 0.24	2.84 ± 0.08	23.10 ± 1.66	14.95 ± 0.77	6.99 ± 0.10	20.49 ± 0.88	21.56 ± 1.29	78.445 ± 1.94
VRSL-1	6.83 ± 0.23	4.62 ± 0.05	21.30 ± 0.89	17.40 ± 0.69	9.76 ± 0.10	16.01 ± 1.26	18.47 ± 0.69	81.53 ± 0.69
VRSL-2	4.53 ± 0.13	4.40 ± 046	26.47 ± 0.90	13.29 ± 0.47	7.33 ± 0.09	20.95 ± 1.42	17.94 ± 0.10	82.055 ± 0.09
VRSL-4	5.14 ± 0.30	3.78 ± 0.04	24.35 ± 1.72	13.73 ± 1.04	6.49 ± 0.09	20.86 ± 1.97	25.24 ± 1.06	74.76 ± 1.05
VRSL-5	4.49 ± 0.18	1.95 ± 0.05	24.44 ± 1.02	17.69 ± 1.33	8.12 ± 0.15	21.69 ± 0.48	22.08 ± 0.59	77.92 ± 0.61
VRSL-6	4.35 ± 0.12	2.88 ± 0.08	24.03 ± 1.54	22.55 ± 1.41	8.95 ± 0.26	20.41 ± 1.20	18.72 ± 1	81.28 ± 1
VRSL-7	5.33 ± 0.13	3.78 ± 0.06	24.45 ± 1.35	19.02 ± 2.73	7.55 ± 0.09	20.35 ± 2.32	19.74 ± 0.26	80.245 ± 0.26
VRSL-8	4.56 ± 0.06	3.42 ± 0.05	24.45 ± 0.93	14.57 ± 0.64	7.35 ± 0.10	22.25 ± 0.80	20.60 ± 0.29	79.405 ± 0.29
VRSL-9	4.62 ± 0.06	3.25 ± 0.07^l^	24.93 ± 1.51	18.92 ± 1.55	8.05 ± 0.01	24.41 ± 1.59	19.79 ± 0.00	80.21 ± 0.01
VRSL-10	5.73 ± 0.12	3.47 ± 0.06	21.84 ± 1.25	19.70 ± 1.43	8.23 ± 0.13	21.53 ± 0.66	21.31 ± 0.04	78.69 ± 0.03
VRSL-11	6.25 ± 0.24	3.16 ± 0.05	23.18 ± 1.60	21.49 ± 1.43	9.45 ± 0.17	21.63 ± 0.69	19.62 ± 0.18	80.395 ± 0.18
VRSL-12	5.81 ± 0.07	4.37 ± 0.06	27.67 ± 0.99	23.32 ± 0.75	7.16 ± 0.11	29.77 ± 0.40	19.76 ± 0.03	75.735 ± 6.36
VRSL-13	6.36 ± 0.13	2.24 ± 0.05	26.11 ± 1.60	18.33 ± 1.35	7.87 ± 0.09	21.33 ± 1.50	21.14 ± 0.21	78.87 ± 0.2
VRSL-14	5.18 ± 0.09	3.10 ± 0.07	24.56 ± 1.86	16.88 ± 2.28	6.82 ± 0.11	22.70 ± 1.46	21.25 ± 0.03	79.365 ± 0.87
VRSL-15	6.28 ± 0.08	3.57 ± 0.06	27.43 ± 0.77	23.63 ± 1.44	7.91 ± 0.13	22.20 ± 1.17	20.0 ± 0.08	81.88 ± 1.54
NDSG-1	5.19 ± 0.06	2.99 ± 0.08	23.78 ± 0.98	17.45 ± 0.85	7.83 ± 0.12	21.92 ± 1.74	22.42 ± 0.30	78.66 ± 1.29
PSG-9	6.49 ± 0.18	3.53 ± 0.06	24.57 ± 1.12^j^	11.91 ± 1.99	9.75 ± 0.10	18.59 ± 1.12	23.17 ± 0.25	76.495 ± 0.7
DSG-31	6.38 ± 0.13	2.36 ± 0.05	20.17 ± 1.23	19.71 ± 1.35	11.54 ± 0.11	15.39 ± 1.40	21.75 ± 0.51	78.24 ± 0.51
CHSG-1	6.22 ± 0.12	2.45 ± 0.05	22.85 ± 1.58	23.61 ± 1.29	11.32 ± 0.20	21.52 ± 0.81	25.31 ± 0.69	74.685 ± 0.7
CHSG-2	6.29 ± 0.06	3.46 ± 0.04	23.75 ± 1.97	26.43 ± 0.80	8.40 ± 0.12	19.78 ± 0.60	25.99 ± 1.83	74 ± 0.82
DSG-43	5.33 ± 0.08	2.87 ± 0.05	22.81 ± 1.87	25.52 ± 0.84	12.18 ± 0.11	16.34 ± 0.89	24.70 ± 0.59	75.285 ± 0.6
DSG-48	6.56 ± 0.14	3.43 ± 0.48	23.22 ± 1.17	15.43 ± 0.81	12.84 ± 0.12	18.45 ± 0.57	24.61 ± 0.47	75.38 ± 0.47
Pusa Sneha	5.75 ± 0.12	2.70 ± 0.05	23.47 ± 0.89	19.43 ± 1.13	8.36 ± 0.07	22.21 ± 0.60	24.40 ± 1.40	75.59 ± 1.4
HASG-5	6.90 ± 0.04	4.28 ± 0.05	24.42 ± 2.44	18.58 ± 0.88	7.13 ± 0.07	19.33 ± 1.92	28.87 ± 5.77	72.46 ± 7.54
PSG-93	6.96 ± 0.06	3.39 ± 0.08	24.99 ± 2.02	17.12 ± 1.58^j^	10.97 ± 0.10	18.88 ± 1.21	22.10 ± 0.53	77.885 ± 0.53
PSG-100	6.50 ± 0.03	2.52 ± 0.05	22.99 ± 1.73	24.29 ± 0.39	11.28 ± 0.12	23.76 ± 1.39	20.96 ± 0.49	78.52 ± 6.67
PSG-110	6.65 ± 0.24	3.43 ± 0.06	24.68 ± 2.73	17.65 ± 1.33	8.46 ± 0.19	20.59 ± 0.78	22.53 ± 0.30	77.455 ± 0.19
NSG-1-11	6.63 ± 0.12	1.25 ± 0.06	24.26 ± 0.78	19.72 ± 1.46	7.54 ± 0.08	20.37 ± 0.68	23.30 ± 0.16	76.695 ± 0.13
NSG-28	6.38 ± 0.06	2.97 ± 0.06	21.11 ± 1.57	16.64 ± 0.75	9.19 ± 0.13	16.87 ± 1.98	22.32 ± 0.14	77.67 ± 0.16
JSLG-55	5.69 ± 0.12	3.71 ± 0.06	21.19 ± 1.43	14.64 ± 1.08	6.43 ± 0.21	22.22 ± 1.59	19.99 ± 0.59	80.005 ± 0.57
DSG-47	5.60 ± 0.06	4.05 ± 0.08	22.52 ± 2.45	16.36 ± 0.91	7.75 ± 0.09	21.13 ± 0.97	21.02 ± 2.33	77.4650.21
Improved Chikni	6.46 ± 0.13	2.34 ± 0.05	21.39 ± 1.16	17.52 ± 1.32	10.25 ± 0.12	15.67 ± 0.95	20 ± 0.18	79.985 ± 0.18
DSG-95	6.57 ± 0.24	3.55 ± 0.64	22.79 ± 0.96	19.57 ± 0.72	10.30 ± 0.07	19.79 ± 0.98	25.50 ± 0.16	74.495 ± 0.16
DSG-98	5.80 ± 0.29	2.16 ± 0.05	24.57 ± 1.47	21.66 ± 2.36	7.44 ± 0.08	21.99 ± 0.75	23.93 ± 0.55	76.05 ± 0.31
DSG-104	6.45 ± 0.24	1.96 ± 0.09	21.67 ± 1.14	14.54 ± 1.35	8.18 ± 0.05	21.87 ± 0.49	22.57 ± 0.25	77.415 ± 0.25^j^
DSG-108	6.64 ± 0.13	2.18 ± 0.06	24.40 ± 1.60	17.8 ± 0.5	7.27 ± 0.06	27.62 ± 1.22	22.97 ± 0.14	77.02 ± 1.12
DSG-26	5.46 ± 0.06	3.24 ± 0.04	25.02 ± 1.33	19.27 ± 0.41	6.87 ± 0.11	27.98 ± 0.63	17.66 ± 0.06	82.345 ± 0.08
DSG-30	6.18 ± 0.07	3.40 ± 0.02	22.35 ± 1.24	21.17 ± 0.13	11.55 ± 0.08	23.72 ± 1.03	21.92 ± 0.06	78.24 ± 0.28
DSG-32	6.02 ± 0.12	2.43 ± 0.07	23.15 ± 1.44	18.91 ± 0.86	12.85 ± 0.07	20.13 ± 1.08	24.08 ± 0.52	75.925 ± 0.53
DSG-34	6.59 ± 0.18	4.11 ± 0.08	24.08 ± 1.68	22.27 ± 0.20	12.22 ± 0.16	24.31 ± 0.51	22.49 ± 0.56	77.52 ± 0.57

Values are means ± S.d.

Total saturated fatty acids—SFAs; Total polyunsaturated fatty acids—PUFAs.

**Figure 2:**
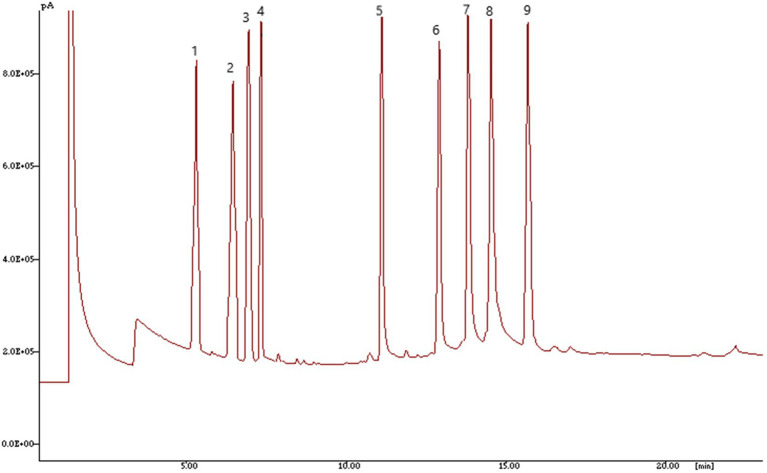
Gas chromatography (GC)-flame ionization detector (FID) profile for FAMES standard mixture done on 30 m HP Innowax column 1—Myristic acid (C14:0); 2—Myristoleic acid (C14:1); 3—Palmitic acid (C16:0); 4—Palmitoleic acid (C16:1); 5—Margaric acid (C17:0); 6—Stearic acid (C18:0); 7—Oleic acid (C18:1); 8—Linoleic acid (C18:2); 9—Linolenic acid (C18:3).

**Figure 3:**
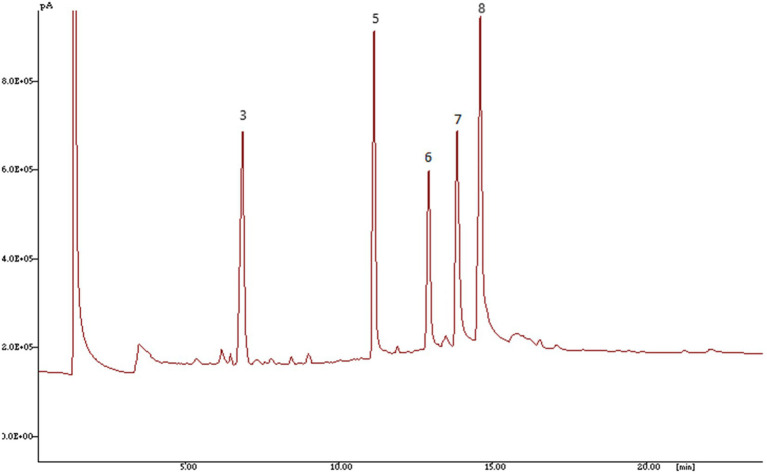
Chromatogram of fatty acids in sponge gourd detected by GC-FID done on 30 m HP Innowax column 3—Palmitic acid (C16:0); 5—Margaric acid (C17:0); 6—Stearic acid (C18:0); 7—Oleic acid (C18:1); 8—Linoleic acid (C18:2).

The standards mixture gas chromatography (GC) chromatogram along with GC chromatogram for genotypes of *Luffa* as determined by GC–MS is depicted in Figures 2, 3, respectively. Linoleic (C18:2), oleic (C18:1), palmitic (C16:0) and stearic (C18:0) are the four major fatty acids present in sponge gourds. Most prevalent fatty acid with a range of 30.86–68.92% was linoleic acid, followed by oleic acid (13.43–45.03%), palmitic acid (10.76–17.75%) and stearic acid (5.93–11.12%; Table 4). The total polyunsaturated fatty acids (PUFAs) of the seed oil ranged from 72.46–82.76% and formed 10 statistically different groups. Total saturated fatty acids (SFAs) had a very low level (17.24–28.87%) with 19 statistically distinct groups. Pusa Supriya (New Delhi) had the highest PUFAs in the seed oil. HASG-5 (Jharkhand) has the highest SFAs concentration.

**Table 4:** Fatty acid composition of 42 genotypes of sponge gourd.

Genotypes	Palmitic acid (%)	Stearic acid (%)	Oleic acid (%)	Linoleic acid (%)
DSG-6	10.76 ± 0.11	8.36 ± 0.32	19.61 ± 0.88	61.28 ± 0.67
Pusa Supriya	11.31 ± 0.22	5.93 ± 0.13	15.56 ± 0.46	67.20 ± 0.80
DSG-7	12.40 ± 0.10	7.87 ± 0.72	39.92 ± 0.33	38.53 ± 1.62
VRSL-1	10.85 ± 0.06	6.07 ± 0.59	19.17 ± 2.43	62.36 ± 3.13
VRSL-2	12.33 ± 0.30	7.09 ± 0.04	16.18 ± 1.20	65.88 ± 1.29
VRSL-4	15.30 ± 0.08	9.95 ± 1.14	34.78 ± 2.78	39.99 ± 3.83
VRSL-5	13.36 ± 0.13	8.73 ± 0.73	31.54 ± 0.64	46.39 ± 0.04
VRSL-6	11.46 ± 0.72	7.26 ± 0.28	18.93 ± 0.65	62.35 ± 1.65
VRSL-7	11.18 ± 0.12	8.56 ± 0.14	16.98 ± 0.59	63.27 ± 0.86
VRSL-8	12.58 ± 0.02	8.02 ± 0.18	27.39 ± 0.55	52.02 ± 0.84
VRSL-9	11.04 ± 0.06	8.75 ± 0.06	19.18 ± 0.78	61.03 ± 0.76
VRSL-10	13.50 ± 0.01	7.81 ± 0.02	25.73 ± 0.37	52.96 ± 0.40
VRSL-11	12.25 ± 0.19	7.37 ± 0.01	19.37 ± 0.34	61.03 ± 0.52
VRSL-12	12.34 ± 0.06	7.43 ± 0.09	19.06 ± 0.78	59.61 ± 2.98
VRSL-13	13.27 ± 0.06	7.88 ± 0.15	24 ± 0.45	54.87 ± 0.65
VRSL-14	13.74 ± 0.04	7.52 ± 0.06	23.57 ± 2.25	55.79 ± 1.92
VRSL-15	11.93 ± 0.43	8.15 ± 0.35	18.48 ± 0.64	63.4 ± 1.92
NDSG-1	14.13 ± 0.42	8.29 ± 0.72	40.95 ± 0.69	37.72 ± 0.60
PSG-9	15.26 ± 0.42	7.91 ± 0.18	36.83 ± 1.05	39.67 ± 0.35
DSG-31	14.75 ± 0.15	7.01 ± 0.36	40.53 ± 0.09	37.72 ± 0.60
CHSG-1	16.37 ± 0.32	8.95 ± 0.37	43.83 ± 0.25	30.86 ± 0.45
CHSG-2	17.66 ± 0.50	8.33 ± 1.33	30.24 ± 1.77	42.26 ± 4.71
DSG-43	17.15 ± 0.17	7.55 ± 0.42	34.6 ± 1.13	43.07 ± 3.90
DSG-48	16.72 ± 0.78	7.90 ± 0.32	36.99 ± 1.63	38.39 ± 1.16
Pusa Sneha	15.60 ± 0.80	8.81 ± 0.6	43.84 ± 0.52	31.76 ± 0.88
HASG-5	17.75 ± 1.21	11.12 ± 1.21	37.91 ± 2.89	34.55 ± 2.95
PSG-93	15.13 ± 0.31	6.97 ± 0.22	34.52 ± 0.86	43.37 ± 1.39
PSG-100	12.99 ± 0.23	7.97 ± 0.26	21.79 ± 0.31	56.31 ± 3.33
PSG-110	14.65 ± 0.24	7.88 ± 0.06	41.48 ± 0.06	35.98 ± 0.25
NSG-1-11	15.86 ± 0.12	7.45 ± 0.28	29.71 ± 0.35	46.99 ± 0.48
NSG-28	15.14 ± 0.06	7.19 ± 0.08	29.41 ± 1.58	48.27 ± 1.42
JSLG-55	11.26 ± 0.22	8.73 ± 0.37	18.87 ± 1.14	61.14 ± 1.71
DSG-47	13.3 ± 1.22	7.72 ± 0.30	30.97 ± 1.37	46.50 ± 1.58
Improved Chikni	11.53 ± 0.13	8.47 ± 0.31	18.06 ± 1.8	61.93 ± 1.62
DSG-95	17.52 ± 0.18	7.98 ± 0.01	43.54 ± 1.43	30.96 ± 1.27
DSG-98	15.74 ± 0.12	8.20 ± 0.43	45.03 ± 0.43	31.03 ± 0.12
DSG-104	14.83 ± 0.01	7.74 ± 0.24	42.26 ± 0.07	35.16 ± 0.18
DSG-108	14.39 ± 0.04	8.58 ± 0.18	35.69 ± 0.66	41.34 ± 0.46
DSG-26	11.24 ± 0.08	6.42 ± 0.14	13.43 ± 0.56	68.92 ± 0.48
DSG-30	13.42 ± 0.05	8.50 ± 0.11	26.76 ± 0.28	51.48 ± 0.0
DSG-32	15.64 ± 0.21	8.44 ± 0.31	39.04 ± 0.89	36.89 ± 0.36
DSG-34	15.19 ± 0.45	7.30 ± 0.11	34.47 ± 0.83	43.06 ± 0.26

Values are means ± s.d.

### 3.3. Correlation analysis

Table 5 displays the correlation between the moisture, ash, protein, oil, sugar, phenol, linoleic acid, oleic acid, palmitic acid and stearic acid that was determined at two distinct significant levels. There was a significant positive correlation between the moisture and oil (0.302, *p* < 0.005 level), the sugar and palmitic acid (0.360, *p* < 0.005 level), the stearic acid and oleic acid (0.338, *p* < 0.005 level). The oil and protein had a strong positive correlation (0.464, *p* < 0.01 level). At the *p* < 0.01 level, palmitic acid exhibited a strong positive association with stearic acid (0.392) and oleic acid (0.796), whereas at the *p* < 0.05 level, it showed a strong negative correlation with linoleic acid (−0.814). The sugar had a statistically significant negative correlation with protein (−0.344, *p* < 0.05 level) and oil (−0.342, *p* < 0.05 level). Linoleic acid showed a strong negative connection with the oleic acid (−0.979) at *p* < 0.05 level and with the palmitic acid (−0.814) at the *p* < 0.01 level. We examined the correlations between longitude, latitude, mean annual temperature (MAT), and the amount of oil, fatty acids, and protein (Table 6). The mean annual temperature was significantly negatively correlated with oleic acid (−0.326, *p* < 0.05 level) and significantly positively correlated with linoleic acid (0.312, *p* < 0.05 level), despite longitude and latitude having no correlation with these quality traits.

**Table 5:** Pearson’s correlation between the traits studied.

	Moisture	Ash	Oil	Protein	Phenol	Sugar	Palmitic acid	Stearic acid	Oleic acid
Ash	−0.048								
Oil	0.302^*^	−0.045							
Protein	0.310	0.105	0.464^**^						
Phenol	−0.099	−0.026	0.197	0.067					
Sugar	−0.058	−0.159	−0.342^*^	−0.344^*^	0.022				
Palmitic acid	−0.082	−0.245	−0.211	−0.067	−0.180	0.360^*^			
Stearic acid	−0.208	−0.074	0.020	0.005	0.106	−0.142	0.392^*^		
Oleic acid	−0.139	−0.374	−0.141	−0.065	−0.204	0.255	0.796^**^	0.338^*^	
Linoleic acid	0.143	0.399	0.147	0.111	0.187	−0.321	−0.814^**^	−0.313	−0.979^*^

*Correlation is significant at *p* < 0.05 (2-tailed).

**Correlation is significant at *p* < 0.01 (2-tailed).

**Table 6:** Correlation of fatty acids, protein, and oil with climatic and geographic variables.

	Oil	Protein	Palmitic acid	Stearic acid	Oleic acid	Linoleic acid
Latitude	0.213	0.233	−0.116	−0.037	0.102	−0.070
Longitude	−0.075	0.151	0.009	0.149	−0.114	0.093
MAT	−0.135	−0.066	−0.216	0.070	−0.326*	0.312*

MAT—Mean annual temperature.

*Correlation is significant at *p* < 0.05 level (2-tailed).

### 3.4. Principal component analysis

In order to further investigate how the traits, contribute to the observed variability and in order to improve the correlation analysis, the principal component analysis (PCA) was carried out to compute the eigenvalues for 10 traits. The findings showed that the first four principal components with eigen values greater than 1 accounted 74.07% of the overall variation among the genotypes (Table 7). The first principal component which was mostly influenced by sugar, palmitic acid, stearic acid, and oleic acid explained 33.58% of the overall variation. The oil, protein, and phenol were primarily related to the second main component, which accounted for 16.87% of the variation. The protein, phenol, stearic acid and linoleic acid made up the third component and explained 12.69% of the variation. While the moisture, oil, phenol, sugar and linoleic acid comprised the fourth component and contributed 10.95% of the variation. According to PCA data, oleic acid, stearic acid, palmitic acid, total phenol, and protein content contributed the most variability among all the attributes being positive in more than one PC.

**Table 7:** Eigenvalues and their proportion for 10 biochemical parameters based on 4 principal components.

Traits	1	2	3	4
Moisture	−0.218	0.166	−0.776	0.042
Ash	−0.439	−0.167	0.327	−0.499
Oil	−0.345	0.746	−0.240	0.195
Protein	−0.258	0.703	0.019	−0.179
Phenol	−0.222	0.155	0.398	0.794
Sugar	0.459	−0.529	−0.181	0.359
Palmitic acid	0.881	0.145	−0.017	−0.091
Stearic acid	0.396	0.409	0.548	−0.023
Oleic acid	0.924	0.231	−0.062	−0.073
Linoleic acid	−0.941	−0.187	0.081	0.021
Total of Eigenvalues	3.36	1.69	1.27	1.10
Percent of total variance	33.58	16.87	12.69	10.95
Cumulative percent of total variance	33.58	50.45	63.14	74.10

### 3.5. Clustering pattern

**Figure 4:**
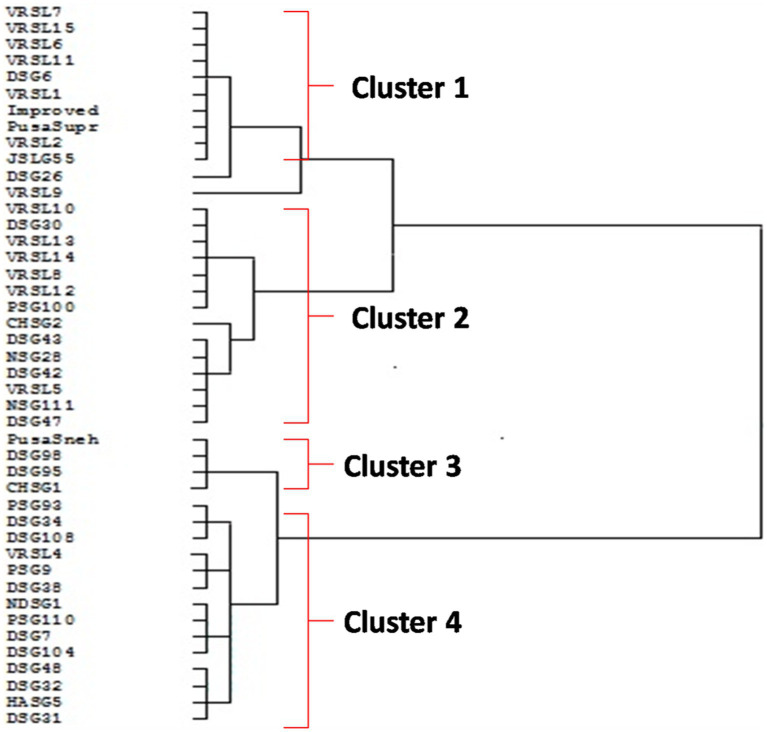
Dendrogram showing relationships among 42 genotypes of sponge gourd based on 10 biochemical characters.

The four clusters were produced based on the Euclidean distance (Figure 4). Cluster I comprised of 10 genotypes (VRSL-7, VRSL-15, VRSL-6, VRSL-11, DSG-6, VRSL-1, Improved Chikni, Pusa Supriya, VRSL-2, and JSLG-55) which showed oleic acid less than 20% and linoleic acid more than 60%. DSG-26 and VRSL-9 were present as outgroups. Cluster II encompassed 14 genotypes (VRSL-10, DSG-30, VRSL-13, VRSL-14, VRSL-8, VRSL-12, PSG-100, CHSG-2, DSG-43, NSG-28, DSG-42 VRSL-5, NSG-1-11 and DSG-47) with 20–31% oleic acid and 45–55% of linoleic acid. There were 4 genotypes in Cluster III (Pusa Sneha, DSG98, DSG95 and CHSG1) which displayed 43.5–45% oleic acid and 30.8–31.8% linoleic acid. Cluster IV comprised of 14 genotypes (PSG-93, DSG-34, DSG-108, VRSL-4, PSG-9, DSG-38, NDSG-1, PSG-110, DSG-7, DSG-104, DSG-48, DSG-32, HASG-5 and DSG-31) which showed 34.5–42.3% of oleic acid, 35–43% of linoleic acid and moderate oil content.

## 4. Discussion

This in-depth biochemical investigation of sponge gourd genetic resources reveals that the nutritional traits under study demonstrate high inter-genotype variability and can be applied to both the industrial and food sectors. The selection and assessment of genetic resources for the intended nutritional qualities are directly related to their use. This is the first in-depth examination of phenolics and fatty acid composition in a broader group of sponge gourds.

According to Chisholm and Hopkins’s research, the oil content of nine species of Cucurbitaceae ranged from 20.1 to 38.3% (46); the oil content of wild and cultivated species of *Luffa* was 25–27% (10). In our study, we found seed oil content in range of 15.39–29.77%. which was in agreement to above researchers. A very low moisture content of 4.35–7.51% was recorded in the genotypes and it is ideal for a long shelf life and less susceptibility to microbial attack. Thus, moisture is a key aspect of food stability and preservation (47). The ash level was found to be between 1.25 and 5.17%, which is consistent with the range of 3.00–5.8% for legumes including cowpea, groundnut, and fluted pumpkin seeds (47). The total amount of minerals in a food can be calculated using the ash value.

This study’s protein content (19.91–30.77%) was much higher than Prakash’s study’s which found that both domesticated and wild sponge gourd species had (8–10%) protein in their seeds (10). The differing approaches taken in the two investigations could be the cause of the discrepancy in protein results. As Lowry’s method, which assesses soluble proteins and is frequently employed when absolute quantities are not required, was used in Prakash’s work (48). Based on the aforementioned findings, the genotypes in our study showed excellent promise as possible sources of protein in the future.

One past investigation reported the protein content of 35.83% in *Luffa cylindrica*, which is slightly greater than what we discovered in our study (47). When the sponge gourd defatted seed’s proximate composition was examined, it was discovered that its protein content ranged from 45.06 to 50.06%, which was higher than that of fatted seeds (49). However, > 25% protein content is considered even more for pulses which consumed as a staple food in several areas of the world (50). The variance might be explained by different ecological factors that have an impact on the plant growth. The primary purpose of nutrition is to provide an adequate amount of amino acids, and proteins are a vital part of the food required for both animal and human survival. Thus, *Luffa cylindrica* could be utilized as a substitute source of protein in diet supplements, especially in regions where the bulk of the population consumes starchy foods and cereals, which serve as the basis for nutrition by providing sufficient amounts of needed amino acids or as a feed additive for cattle (51).

Total polyunsaturated fatty acids (PUFAs) content was determined to be 72.46–82.76%, while total saturated fatty acids (SFAs) content was found to be 17.24–28.87% in our study. However, prior study reported 52.02% of PUFAs and 33.07% of SFAs and in seeds of *Luffa cylindrica* (52). The sponge gourd’s fatty acid composition was found to be comparable (palmitic 10.76–17.75%, stearic 5.93–11.12%, oleic 13.43–45.03% and linoleic of 30.86–68.92%) to oilseed crop Niger (palmitic 5.8–3.0%, stearic 5.0–7.5%, oleic 13.4–9.3%, and linoleic of 45.4–65.8%) (53). There was a little change in the trend of the fatty acids profile in the study conducted by Lucy and Abidemi (52) in sponge gourd seeds, where palmitic acid and stearic acid content were found to be 12.86 and 15.17% similar to our results, but the percentage of oleic acid (2.57%) and linoleic acid (31.47%) is much lower. Genotypes with oleic acid up to 45% is a novel finding in our research as compared to earlier reports (46, 52).

One of the most important unsaturated fatty acids known to serve a critical role in human nutrition is oleic acid. Oils with a high oleic acid content are highly resistant to heating and oxidation. Therefore, high oleic acid oils can be used in place of saturated fats in food service applications requiring long-life stability because it has been demonstrated that they exhibit heat stability that is equivalent to saturated fats (54). The most prevalent fatty acid found in the sponge gourd seeds was linoleic acid. It affects blood lipid levels favorably and is connected to a lower risk of coronary heart disease. Since linoleic acid and any of its derivative fatty acids cannot be produced by humans, they must be obtained through diet. The significant reduction in atherosclerosis caused by the high linoleic acid content and both linoleic and oleic acids lower blood cholesterol levels (55).

The results of this study showed that genotypes having the greatest levels of oleic acid and linoleic acid can be used for genetic testing and breeding initiatives. We discovered that the genotypes NDSG-1, Pusa Sneha, DSG-95, DSG8, and DSG108 had higher oil yields (> 105 Kg ha^–1^), high oleic acid levels (> 35%), and high protein levels (25% or more). A high oil output, protein, and linoleic acid (68.9%) were found in the DSG-26 genotype, although it had a low oleic acid content (13.4%). Therefore, these genotypes of sponge gourd could be exploited as a good source of oil and protein.

We found no association of the latitude and longitude with the oil and fatty acids. Yunxia Ma also discovered no connection between longitude and the 26 provenances of *Xanthoceras sorbifolium* Bunge (56). Nevertheless, the oil content of *Helianthus annuus* L. seeds showed significant positive correlation with longitude and latitude (57). Temperature is one of the primary factors that affects linoleic acid and oleic acid content in oilseed crops. MAT was observed to be significantly negatively correlated with oleic acid and significantly positively correlated with linoleic acid. Several researchers reported the significant negative association between low temperature and amount of oleic acid present for example, in maize and soybean; sunflower (58, 59). It is stated that seed oils of plants grown in cool climates tend to be more unsaturated than those grown in warm climates. This might be due to increase in accessible oxygen, which was the rate-limiting element for desaturation, and thus increase in the level of unsaturated fatty acids in seeds at low temperatures (60). However, different plant species’s fatty acids showed different response to temperature (60). A genetic correlation between traits is produced via pleiotropy and linkage disequilibrium, which prevents traits from varying independently of one another (61). Because choosing one trait indirectly chooses a genetically associated quality, correlation studies are helpful for selecting varieties with enhanced composition (62). Significant positive relationship was observed between oil and protein and both were significantly negatively correlated with sugar, respectively. Therefore, utilizing such traits in breeding programs will be beneficial and contribute to improved varietal development for use as a replacement oilseed crop. Consequently, selecting genotypes with a high oil content and high protein is possible as such features which shows significant relations are controlled by tightly linked genes. It was discovered that linoleic and oleic acids were negatively associated, and other oilseed crops, including crucifer species, soybean, peanut, sesame, and safflower, also had this type of linkage (45, 63, 64).

A crucial tool in the parent-selection process is the measurement and classification of genetic variation between genetic materials. PCA and cluster analysis are practical statistical techniques that complement one another effectively for this objective. While PCA is used to assess the variability’s magnitude, cluster analysis is used to categorize the variability. The clustering of genotypes based on their genetic similarity facilitates the discovery and choice of the ideal parents for specific breeding operations (65). The PCA revealed that factors like oleic acid, stearic acid, palmitic acid, total phenol, and protein content were responsible for the majority of the difference between genotypes. The clustering pattern produced four clusters, but they were largely clustered according to the fatty acid profile rather than the place where the seeds were collected.

The Indian subcontinent is rich in genetic variety in cucurbits, with domesticated, semi-domesticated, or wild species occurring in small pockets (66). In addition to fruits, as a vegetable it can also play a significant part in ensuring nutritional security by utilizing the seeds which are a great source of protein and oil. Its commercial use offers great potential for diversifying the types of vegetables that can be farmed. Sponge gourd has got wider adaptability and is found to grow in semi-arid to high rainfall areas. Moreover, there is considerable tolerance in sponge gourd to biotic and abiotic stresses and the crop requires nominal outside efforts leading to the small cost of cultivation and high economic returns (67).

## 5. Conclusion

Our findings show that there is large variability in sponge gourd genotypes for biochemical parameters, and these variations have a tremendous potential to be used to create genotypes with improved oil quality and nutrient content. This study found the genotypes (NDSG-1, Pusa Sneha, DSG-95, DSG-98, DSG-26 and DSG-108) with the greatest number of good nutritional characteristics like oil yield, high oleic acid and protein; consequently, choosing such genotypes for creating new crosses in breeding would be helpful. Additionally, the nutritional value, fatty acid composition, and phenolic content of sponge gourd seeds enable for both their use as an oil source for industrial purposes as well as a dietary food supplement to meet the nutritional demands of developing nations. As a result, NDSG-1, Pusa Sneha, DSG-95, DSG-98, DSG-26 genotypes of sponge gourd can be used as a nutritious vegetable, while DSG-10 high oil yield can be used to make edible or industrial oil. More research is required before sponge gourd seeds are recommended for human nutrition.

## Data availability statement

The raw data supporting the conclusions of this article will be made available by the authors, without undue reservation.

## Author contributions

MV and LA conceived and designed the experiment. RT performed the experiment and drafted the manuscript. RB analyzed the data and revised the manuscript. PS assisted or supported in GLC operation. AM and AS provided the seed samples of sponge gourd inbred lines. All authors contributed to the article and approved the submitted version.

## Funding

This work was funded by project Consumption of Resilient Orphan Crops and Products for Healthier Diets (CROPS4HD) which is financially supported by the Swiss Agency for Development and Cooperation, Global Programme Food Security (SDC GPFS) and executed in India through FiBL (Research Institute of Organic Agriculture).

## Conflict of interest

The authors declare that the research was conducted in the absence of any commercial or financial relationships that could be construed as a potential conflict of interest.

## Publisher’s note

All claims expressed in this article are solely those of the authors and do not necessarily represent those of their affiliated organizations, or those of the publisher, the editors and the reviewers. Any product that may be evaluated in this article, or claim that may be made by its manufacturer, is not guaranteed or endorsed by the publisher.
